# Effect of epiretinal electrical stimulation on the glial cells in a rabbit retinal eyecup model

**DOI:** 10.3389/fnins.2024.1290829

**Published:** 2024-01-22

**Authors:** Dean Henze, Joseph A. Majdi, Ethan D. Cohen

**Affiliations:** ^1^University of San Diego, San Diego, CA, United States; ^2^Division of Biomedical Physics, Office of Science and Engineering Labs, Center for Devices and Radiological Health, Food and Drug Administration, White Oak Federal Research Labs, Silver Spring, MD, United States

**Keywords:** Müller cell, rabbit retinal eyecup, glutamine synthetase, microglia, optical coherence tomography, electrical stimulation, transparent electrode

## Abstract

**Introduction:**

We examined how pulse train electrical stimulation of the inner surface of the rabbit retina effected the resident glial cells. We used a rabbit retinal eyecup preparation model, transparent stimulus electrodes, and optical coherence tomography (OCT). The endfeet of Müller glia processes line the inner limiting membrane (ILM).

**Methods:**

To examine how epiretinal electrode stimulation affected the Müller glia, we labeled them post stimulation using antibodies against soluble glutamine synthetase (GS). After 5 min 50 Hz pulse train stimulation 30 μm from the surface, the retina was fixed, immunostained for Müller glia, and examined using confocal microscopic reconstruction. Stimulus pulse charge densities between 133–749 μC/cm2/ph were examined.

**Results:**

High charge density stimulation (442–749 μC/cm2/ph) caused significant losses in the GS immunofluorescence of the Müller glia endfeet under the electrode. This loss of immunofluorescence was correlated with stimuli causing ILM detachment when measured using OCT. Müller cells show potassium conductances at rest that are blocked by barium ions. Using 30 msec 20 μA stimulus current pulses across the eyecup, the change in transretinal resistance was examined by adding barium to the Ringer. Barium caused little change in the transretinal resistance, suggesting under low charge density stimulus pulse conditions, the Müller cell radial conductance pathway for these stimulus currents was small. To examine how epiretinal electrode stimulation affected the microglia, we used lectin staining 0–4 h post stimulation. After stimulation at high charge densities 749 μC/cm2/ph, the microglia under the electrode appeared rounded, while the local microglia outside the electrode responded to the stimulated retina by process orientation inwards in a ring by 30 min post stimulation.

**Discussion:**

Our study of glial cells in a rabbit eyecup model using transparent electrode imaging suggests that epiretinal electrical stimulation at high pulse charge densities, can injure the Müller and microglia cells lining the inner retinal surface in addition to ganglion cells.

## 1 Introduction

When stimulation electrodes are placed on the inner retinal surface such as in an epiretinal prosthesis, the cell types often closest to the stimulus electrode are the processes of glial cells. After the inner limiting membrane (ILM), the endfeet processes of the Müller glia cell are often the closest cellular elements to epiretinal electrodes, ensheathing the ganglion cell bodies. It is generally believed that the strongest electric fields are thought to be developed where the electrodes are in close proximity to the retinal tissue (e.g., Shannon, 1992; Minnikanti et al., 2010). As a consequence of their inner retinal location, Müller glia, astrocytes, and microglia are the three main types of glial cells that have the potential to experience strong electric fields during epiretinal electrical stimulation. Thus epiretinal stimulation electrodes, in addition to being near retinal neurons, are also in close contact with the processes of retinal glia cells which could cause cellular injury.

Glial cells play key roles in supporting retinal homeostasis. The endfeet processes of Müller (macro) glia form a semi-permeable retinal barrier next to the ILM. Müller cell retinal homeostatic roles include neurotransmitter recycling, ion homeostasis, glia-neuron communication, glutamate metabolism, maintenance of the blood-retinal barrier, parafoveal cone light guides, and energy provision (Labin et al., 2014). These functions are particularly important to stabilize the neurons in patients with retinal degenerations such as retinitis pigmentosa. Transporters in the Müller glia cell control extracellular levels of neurotransmitters such as the excitatory amino acid glutamate and GABA (Bringmann et al., 2013). Neuronal waste products ions such as ammonia, and potassium, are removed from the interstitial space by Müller glia cells (Kofuji and Newman, 2004; Kuo et al., 2020), while pyruvate is supplied to retinal neurons. While Müller cells express many voltage-gated channels, large inward rectifier conductances to potassium ions and leak channels are often found on their end feet processes (e.g., Solessio et al., 2000; Newman, 2005; Hughes et al., 2017). The high conductance of inward rectifier potassium channels on Müller glia are thought to help maintain the ion homeostasis of the inner retina (Newman, 1986; Chao et al., 1994). Astrocytes are homeostatic (macro) glial cells that help direct retinal vasculature development (Paisley and Kay, 2021) and are found at the optic radiations similar to oligodendrocytes (Morcos and Chan-Ling, 1997). Finally, retinal microglia cells are *responsible for* immune surveillance, synaptic pruning, retinal vascularization, and the removal of dying cells (Colonna and Butovsky, 2017; Fan et al., 2022; Murenu et al., 2022).

We have developed a clinically relevant *ex-vivo* rabbit retinal eyecup model preparation where the live retina remains attached to the retinal pigment epithelium for use with optical coherence tomography (OCT). This allows optical imaging of the whole eye-wall using transparent stimulus electrodes similar in size to those used in retinal prostheses (Cohen et al., 2011). By using this merangiotic retina model preparation, a saline-filled optically transparent fluoropolymer tube as a stimulation electrode and OCT, we are able to position stimulus electrodes in fluid at a defined proximity from the inner retinal surface and image the damage to the retinal layers by high charge density electrical pulse trains in real-time (Cohen, 2009; Cohen et al., 2011). Using the rabbit retinal eyecup model and OCT, we observed that high charge density pulse train electrical stimulation caused a detachment of the inner limiting membrane from the underlying ganglion cell layer (GCL) under the electrode, and swelling in the inner retina. This retinal layer in rabbit is thought to contain many Müller glial cell endfeet, microglia and ganglion cells. In addition, during inner retinal surface stimulation at high pulse charge densities (442–749 μC/cm^2^/ph) at 50 Hz, OCT cross sectional B-scans showed an edge-like pattern of photoreceptor detachment was seen in the outer retina in the subretinal space directly below the edges of the stimulus electrode (Cohen et al., 2011). We previously showed pulse train stimulation at these high charge densities (442–749 μC/cm^2^/ph) could abolish the ganglion cell light-responses for many minutes, (i.e., Cohen, 2009), and induce retinal edema and injury in ganglion cells (Cohen et al., 2011). Histology analysis of these stimulated retinas suggested that these high charge density pulse trains may also damage glial cell processes. Thus, we decided to examine how epiretinal electrical stimulation of the inner retinal surface could affect the glial cells, particularly the Müller cells, which are reported to have limited regenerative capacity in humans (Gao et al., 2021; Salman et al., 2021).

## 2 Methods

### 2.1 Preparation

The rabbit eye wall was prepared for retinal imaging by using a modified superfused rabbit retinal eyecup preparation (see Miller et al., 1986; Cohen et al., 2011 for details). Twenty one rabbits (either sex) were anesthetized with ketamine-xylazine (35–50 mg/kg^−1^, 5–10 mg/kg) followed by pentobarbital euthanasia using a protocol approved by the FDA animal research committee. An eye was removed, hemisected, and the posterior half of the eye-wall containing the retina was everted on a Teflon dome, and covered with an insulated eyecup recording chamber. The eyecup chamber was superfused with an oxygenated (95% O_2_/5% CO_2_) bicarbonate-buffered Ames Ringer (Sigma Chem, St Louis, MO or US Biological, Swampscott, MA). It contained the following salts (in mM): 120 NaCl, 3.1 KCl, 0.5 KH_2_PO_4_, 23.0 NaHCO_3_, 1.2 Mg_2_SO_4_, 1.15 CaCl_2_, phenol red indicator, and 26 vitamins and amino acids. The solution was heated to physiological temperature (34°C–35°C) and flowed across the preparation at a rate of 5–6 mL/min. The surface area of the rabbit eyecup chamber model is large (1.13 cm^2^, 12 mm diam.) which reduces animal use by allowing multiple test stimulation zones on the retinal surface. The low chamber volume of 350 μL allows good oxygenation and rapid drug exchange rates.

### 2.2 OCT and fundus imaging

A custom-made Fourier domain OCT unit (Physical Sciences Inc., Andover, MA) was used to perform the retinal imaging, and position the electrode. Experiments were performed on a mesopic background light of ~70 lux (see Cohen et al., 2011). The scan beam was focused onto the retinal surface of the eyecup chamber using an achromat and mirror. A glass coverslip was placed over the eyecup preparation fluid to improve retinal imaging. Time lapse OCT images were collected as a series of 20 1K × 1K B-scan images at 13 Hz, averaged and stored as 8-bit serial bitmap images (see Cohen et al., 2011 for details). A scanning line ophthalmoscope channel allowed gross viewing in the eyecup chamber of the retinal location of the stimulating electrode tube tip near the retinal surface (which was faintly visible), and the optic nerve head. An eyecup image was recorded for each stimulation electrode retinal location. Eyecup preparations were stimulated with high-level current pulse train electrode stimulation in different retinal locations, followed by another 5–15 min imaging post-stimulation. In the OCT image, the retina remained attached to the pigment epithelium in each eyecup preparation for >3.5 h of Ringer superfusion allowing for local study of multiple stimulation zones.

### 2.3 Stimulation

**Figure 1:**
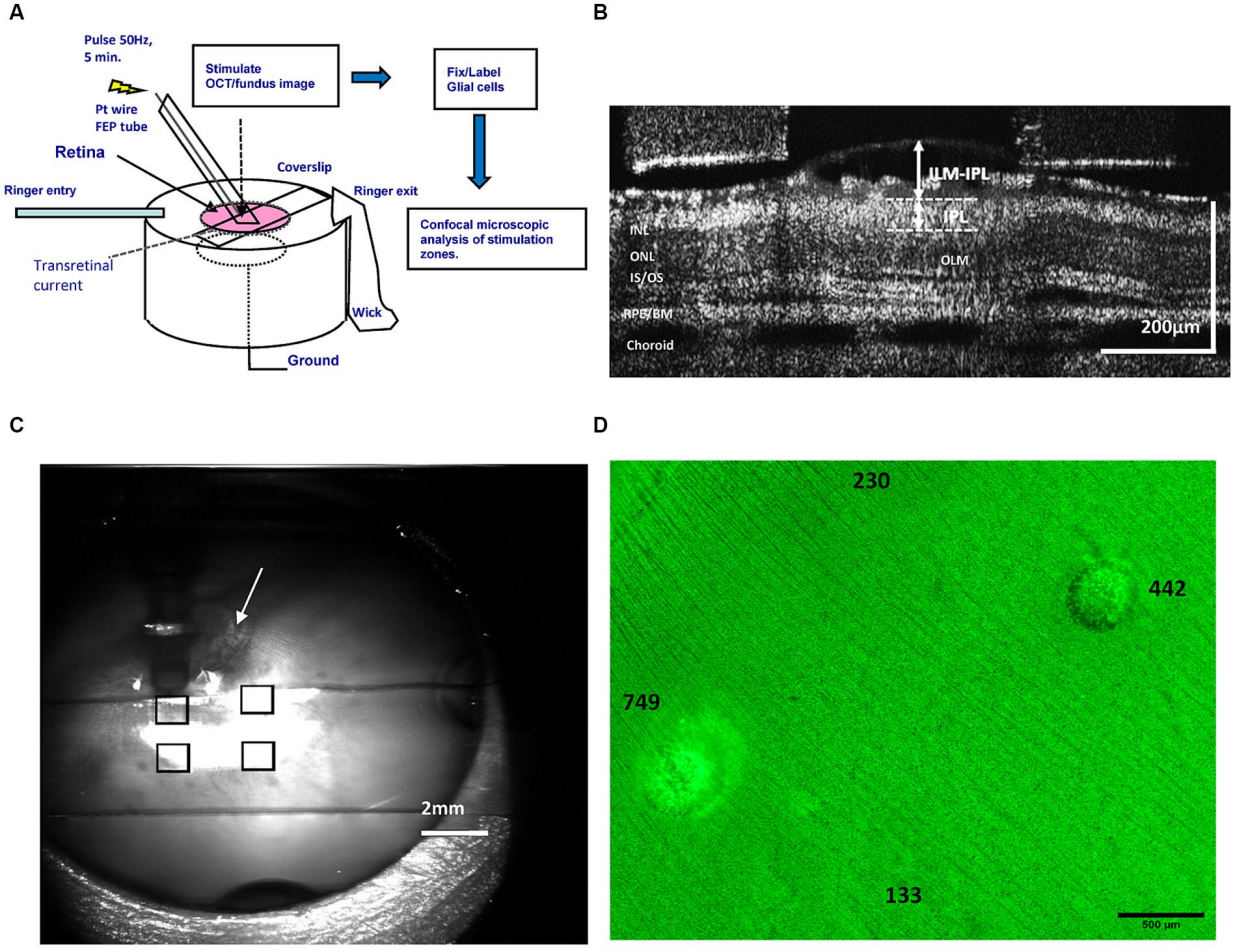
**(A)** Diagram of the epiretinal electrode stimulation, current injection, optical imaging, and retinal tissue image processing methods performed using the rabbit retinal eyecup preparation model and an FEP Teflon transparent stimulus electrode. **(B)** Method of OCT B-scan layer measurements of the inner retina during stimulation at different pulse charge densities. We used OCT to measure swelling in the GCL by ILM-IPL distance (arrows). **(C)** Method of mapping of stimulation-evoked lesions on the retinal surface by superposition of transparent electrode stimulation positions in the ophthalmoscope (eyecup) image. The stimulus tube electrode pointing into the fluid is the dark vertical object seen above the upper left square. By superimposing ophthalmoscope images of each electrode position (squares), a retinal stimulation location map could be generated. Arrow denotes optic nerve head used as an alignment landmark. **(D)** Epifluorescence microscope image montage of a GS-stained retinal wholemount stimulated with pulse trains of different charge density at the indicated locations (numbers). After stimulation, the Müller glia were labeled green with antibodies against GS, and damage appears as ring-like lesions post-stimulation. The number denotes the pulse charge density μC/cm^2^/ph of the pulse trains applied by the stimulus electrode at that location on the retinal surface. ILM, inner limiting membrane; OLM, outer (external) limiting membrane; IPL, inner plexiform layer; INL/ONL, inner/outer nuclear layer; IS/OS, inner/outer segment junction; RPE/BM, retinal pigment epithelium/Bruch’s membrane.

Biphasic stimulus current pulses, 1 msec/phase at 50 Hz were delivered to the retina through a platinum wire in a short saline-filled transparent fluoropolymer tube faced at a 45° angle to the retinal surface (Figure 1). Retinal stimulation was performed at an average electrode proximity of 29.9 ± 4.5 μm (mean ± s.d. *n* = 24 zones) from the retinal surface. This electrode proximity was used to avoid direct physical damage or anoxia altering the retinal structure of the superfused eyecup preparation in OCT B-scans. Optically transparent Teflon FEP tubing (*n* = 1.338) (Zeus Inc., Orangeburg, SC) 0.38 mm i.d., 0.89 mm o.d. was used for all the experiments. The cut face of the tube was carefully aligned to be flush to the retina surface using a Sutter Inst. MP285 micromanipulator (Novato CA) and a tilting chamber platform. Biphasic cathodic-anodic pulses (132, 230, 442, or 749 μC/cm^2^/ph) were generated using an AM systems Model 2300 Digital Stimulus isolator (Everette, WA) under computer control using a Micro 1401 data acquisition/control unit and the Spike 2 programming language (Cambridge Electronic Design, Cambridge, United Kingdom) (see Cohen et al., 2011 for details).

The stimulation was delivered by the transparent electrode tube which functioned as a salt bridge across the eyecup layers for a period of 5 min. A Pt–Ir foil 13 mm in diameter lined the eyecup chamber bottom which functioned as a diffuse ground electrode. The pulse train waveform, frequency, charge densities, and duration (50 Hz, 1 msec/phase) tested were similar to those used in other studies of prosthetic electrical stimulation injury (e.g., McCreery et al., 1990, Butterwick et al., 2007; Nakauchi et al., 2007; Cohen, 2009; Cohen et al., 2011, see also Discussion). A total of 24 stimulation zones were analyzed, with 4–5 zones studied per eyecup.

### 2.4 Immunocytochemistry: Müller cells and astrocytes

We performed immunocytochemistry to label the glial cells in the stimulated retina zones in the eyecup to compare to the same zones seen in retinal OCT B-scan cross-sectional images. To help register the OCT/fundus images of the stimulated retinal zones in each eyecup experiment to the same areas after post processing immunocytochemistry, a high stimulation zone was always included (442–749 μC/cm^2^/ph) which our previous OCT stimulation studies showed to induce retinal damage. The eye-wall was carefully removed from the recording chambers and fixed in 4% paraformaldehyde in phosphate buffer for 1–2 h. After fixation, the isolated retinal eyecup sheets were rinsed in 0.1 M phosphate buffered saline (PBS), and incubated in 10% normal goat serum in PBS in 1% Triton X-100 (Sigma) 2 h at room temperature. To label the Müller cells acutely, we used an antibody against the soluble enzyme GS (also termed glutamate-ammonia ligase) (Riepe and Norenburg, 1977). Samples were PBS rinsed and incubated in either mouse anti-GS antibody diluted 1:250 by volume in PBS with 0.3% Triton X-100 for 3 days at 4°C on a shaker. For astrocytes, the retina was incubated with mouse anti-glial fibrillary acid protein (GFAP) antibodies BD # 556330 (Franklin Lakes, NJ). After 5 PBS rinses, each retina was incubated in 1:200 goat anti-mouse secondary antibodies using DiLite fluorophores (Jackson Immunoresearch, West Grove, PA). Control stains of the retina in the secondary antibody alone showed little or no fluorescence signal. Tissue samples were rinsed in PBS, counterstained with DAPI, and mounted on coverslipped slides using FluoroGel mounting medium (EMS, Hatfield PA). The fluorescent tissue stimulation zones of the retinal sheet were then photographed as a montage at 2.5× on a Zeiss Axioplan light microscope (C. Zeiss, White Plains, NY) using a Canon EOS20D digital camera. Stimulated zone montages were reconstructed from the photomicrographs using the free Image Composite Editor (ICE) (Microsoft, Redmond WA).

### 2.5 Microglia lectin labeling

We first tested prelabeling the microglia by injecting a dye-coupled lectin into the eye of the rabbit. Anesthetized rabbits were intravitreally injected with 100 μL Alexa 488 Griffonia Isolectin B4 in Ames Ringer, (Invitrogen, Carlsbad CA). In the rabbit this lectin labels retina microglia and a few blood vessels in the optic radiations. After 1 hr, the eye was enucleated as previously described and the retinal eyecup was imaged on a confocal microscope using a water immersion 40× lens in Ames Ringer. However this microglia field staining method often resulted in patchy labeling of the microglia processes with time after incubation, so we chose to bath incubate the retinal eyecup in the lectin post-stimulation.

We studied the morphological changes in the response of the microglia to high pulse charge density electrical stimulation of the rabbit retina at different times post-stimulation. Each stimulation location was stimulated at 749 μC/cm^2^/ph, 50 Hz, for 5 min at 35°C in the eyecup chamber at the same close retina proximity, however individual locations received stimulation at different times (up to 4 hr post stimulation). To uniformly label the microglia post stimulation, the rabbit retinal eyecup was incubated in a fluorescent red DyLight 594 dye-conjugated Griffonia Isolectin B4 25 μg/mL in Ames Ringer at 25°C (Vector Labs, Burlingame CA) (see Uckermann et al., 2005), using gassed recirculation as microglia motility is low at room temperature (Seo et al., 2012). The eyecup was then washed in Ames Ringer, and fixed in 4% paraformaldehyde. The isolated retina was mounted for confocal microscopy using the blue fluorescent dye DAPI as the nuclear counterstain. Retinal Z-stacks were flattened using the FIJI MAX function for analysis of lesions. An ellipse was drawn around the lesion edge. A series of 100 μm diam. circular ROIs were drawn around the edge and the inner microglia in each circle were analyzed for orientation and coherency using the FIJI OrientationJ plug-in relative to the central lesion axis.

### 2.6 Confocal microscopy and image analysis

Immunostained retinal zones showing glial cell lesions were imaged post stimulation using an Olympus Fluoview 1,000 confocal microscope at 10× magnification using Z-stack image reconstruction. Measurements of swelling and stimulated zones were performed using FIJI-based macros (Schindelin et al., 2012). In OCT B-scan images, swelling was measured pre stimulus (*t* = 5 min), and 5 min post stimulus (*t* = 15 min). Swelling was quantified as the distance between the inner IPL border and the inner limiting membrane (ILM) in the OCT image. In rabbits, this GCL zone normally contains mainly Müller cell endfeet plus ganglion and displaced amacrine cell bodies (Oyster et al., 1981). IPL thickness changes were measured in a similar manner. In isolated retinas immunostained for GS, the area outline of the overstimulated test stimulus zones of hypofluorescence were measured in the fluorescent microscope photomontages by drawing polygons in FIJI.

### 2.7 Eyecup bioimpedance measurements

**Figure 2:**
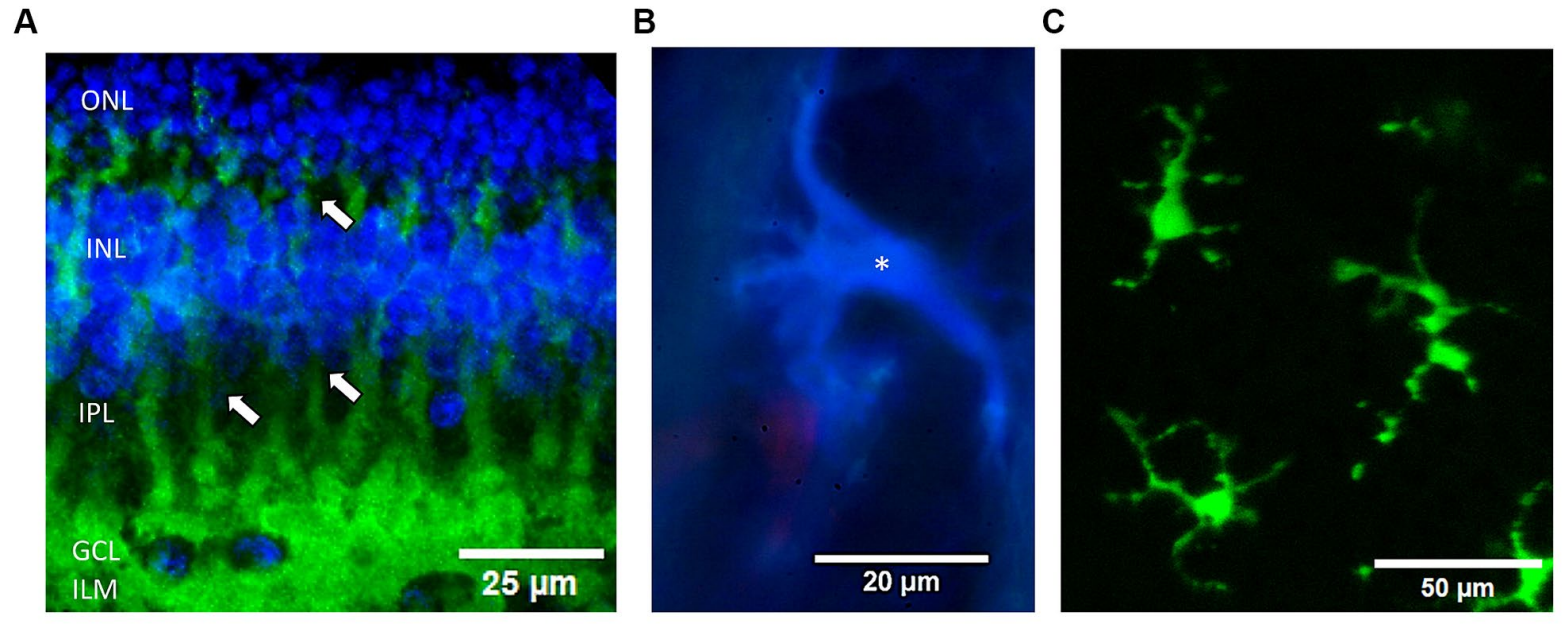
Examples of the 3 glial cell types examined in the rabbit retina. **(A)** Müller cells are immunolabeled by antibodies against GS (green) from a confocal microscope Z-stack. DAPI was used to label nuclear layers blue. Note the processes (arrows) of individual Müller cell endfeet form a near continuous border at the inner retinal surface (bottom) and envelop the ganglion cells (blue nuclei). ONL, outer nuclear layer; INL, inner nuclear layer; IPL, inner plexiform layer; GCL, ganglion cell layer; ILM, inner limiting membrane, respectively. **(B)** Fluorescence micrograph of an astrocyte immunostained with anti-GFAP (blue) (*) in the optic nerve radiations near a red Griffonia lectin labeled blood vessel. **(C)** Live fluorescent lectin-labeled microglia cells (*) from a summed confocal microscope Z-stack image. Cells were prelabeled in the eye with Griffonia isolectin B1 (green).

To measure the changes in transretinal eyecup resistance during application of channel blockers, we generated transretinal current pulses across a modified eyecup preparation by using two large and ring-shaped Pt–Ir wire electrodes placed on either side of the eyewall, a WPI A395 (Sarasota, FL) linear stimulus isolator under computer control (pClamp, Axon Instruments, Novato CA). Currents passed from the Pt–Ir electrode in the bath through the eye-wall layers through a porous saline-filled domed frit to the Pt–Ir counter electrode (Figure 1A). The eyecup tissue charging voltage waveform to the current pulse was recorded using a Ringer-filled AgCl glass electrode (2–4 MΩ) positioned near the retinal surface and a DAGAN 3900A patch clamp amplifier in current clamp mode (Dagan Corp, Minneapolis, MN) referenced to an AgCl ring ground electrode located just behind the eyecup tissue. Data were sampled at 25 kHz using a Digidata 1440A data acquisition system (Molecular Devices, Sunnyvale, CA). Retinal charging voltage waveforms were analyzed using a two time constant method of fit (see Figure 2 for details). Curve fitting of retinal charging voltage waveforms was performed using Origin software, Microcal (Northampton, MA).

### 2.8 Statistical tests

Statistical tests were performed with the appropriate paired *t*-test functions in MS Excel, for normally distributed data, or for non-normally distributed data (Kruskal Wallis test), using the SOFA (Statistics Open For All) statistics package (Paton-Simpson & Associates Ltd., Aukland NZ).

## 3 Results

**Figure 3:**
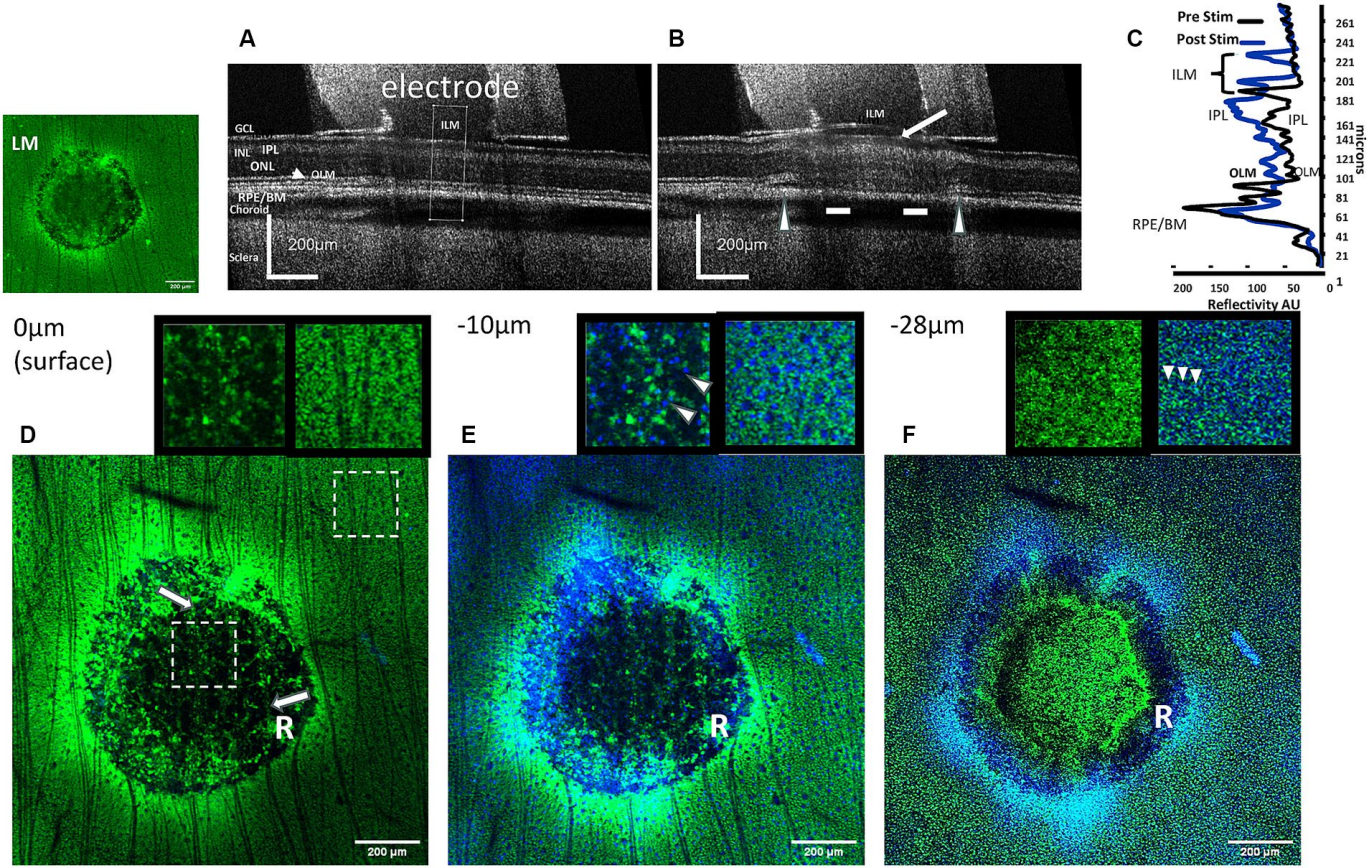
Comparison of OCT images of the effects of high charge density electrode stimulation of the retina with confocal images of immunolabeled Müller cells at several depth levels in the stimulation zone. **(A)** OCT B-scan cross sectional image of the retina unstimulated (control). The cross-sectional analysis zone is seen in the middle of the tube (dotted lines). **(B)** OCT B-scan cross sectional image of the post-stimulated retina at a high charge density (749 μC/cm^2^/ph) causes ILM detachment and formation of bleb (arrow). In the outer retina, at the electrode edges the retina became detached (arrowheads), while the retinal layers are less distinct (bars). **(C)** Comparison of the OCT retinal layer reflectivity profiles before and after stimulation in the analysis zone. The ILM is displaced due to detachment from the GCL (curly arrows). Large changes in reflectivity can be seen in the IPL of the inner retina, and smaller changes in the outer retina. LM: fluorescence micrograph of GS-immunostained (green) stimulated pulse train retinal zone lesion. **(D)** Confocal GS-immunostained fluorescent section of the same stimulated retinal zone at the inner retinal surface (0 μm). In the unstimulated retina surrounding the zone, the inner retinal surface is covered with labeled Müller cell endfeet and nerve fibers. In the electrode stimulated zone, note the absence of GS fluorescence in the center area, and an outer edge ring of disrupted fluorescence (R). Only, ganglion cell nerve fibers (arrows) course over the stimulated retinal zone. Insets: Shows enlarged 200 × 200 μm electrode stimulated (right) and unstimulated (left) zones (dashed box locations). Unstimulated area shows block-like GS immunostained Müller cell endfeet (green) absent in the stimulated zone under electrode. **(E)** Confocal image 10 μm below the inner retinal surface of the stimulated zone. Tiny DAPI-stained nuclei (blue) of retinal ganglion cells (arrowheads) can be seen throughout the lesion center, however GS stain of Müller cell endfeet is absent. Inset: unstimulated and stimulated areas 2× (same location as **D**). **(F)** Confocal micrograph 28 μm below the inner retinal surface near the inner nuclear layer of the stimulated zone. At this level the processes of the Müller cells are seen as numerous fine dots in the retina surrounding the zone, while they are disrupted in the stimulated zone. Note in the stimulated zone a GS-negative dark ring is present near the electrode edge (R). Some diffuse GS positive staining can now be seen in the center of the stimulated zone. ILM, inner limiting membrane; GCL, ganglion cell layer; IPL, inner plexiform layer; ONL, outer nuclear layer; OLM, outer (external) limiting membrane; RPE/BM, retinal pigment epithelium/Bruch’s membrane.

The experimental paradigm for the testing for glial cell injury produced by different levels of electrical stimulation is shown in Figure 1 using the rabbit retinal eyecup model preparation. We used time-lapse cross sectional OCT B-scans to image how stimulation affected the retinal structure at different charge densities of 50 Hz pulse trains. To examine the effects of different levels of electrical stimulation on the underlying retina, biphasic stimulus current pulses at either 133, 230, 442, or 749 μC/cm^2^/ph were applied at 50 Hz for 5 min at discrete locations near the inner limiting membrane using the transparent stimulation electrode. We imaged each electrode stimulus location on the retinal eyecup using the ophthalmoscope camera channel to form a montage map as shown in Figure 1C. Stimulus locations were spaced ≥1,500 μm apart. The retina wholemount was then fixed and processed for immunocytochemistry against glutamine synthetase (GS) a soluble enzyme found in Müller glial cells (Riepe and Norenburg, 1977). An example of a light microscope GS immunofluorescence montage of a retinal wholemount showing a series of sequentially stimulated zones from a single experiment is shown in Figure 1D. Stimulation zones on the retinal surface are mapped to their respective stimulation areas and the pulse charge density delivered. Pulse charge densities were examined both above and below those known to produce retinal tissue swelling and scatter in OCT images (Cohen et al., 2011). The retina of these stimulation areas would then be compared microscopically to examine if a stimulation level altered the structure of the underlying glial cells. The rabbit retina contains principally 3 different types of glia cells (Figure 3). We chose selective glial specific antibodies that would label these cell types acutely after the injury as the preferred pathological analysis method (Switzer, 2011). DAPI dye was often applied as a fluorescent blue nuclear counterstain.

### 3.1 Müller cells

Antibodies against GS were able to acutely label Müller cell processes from their external junctions in the outer retina to their endfeet at the ILM (Figure 2A). Because the endfeet processes of the Müller cells are known to form a near continuous surface facing the ILM (Halfter et al., 2014) we first investigated if acute injury of the Müller cell end feet and loss of soluble GS immunofluorescence might be a good indicator of excessive stimulation injury damage by epiretinal electrodes at high charge densities. We were interested in acute effects on the Müller cells as as our previous OCT study of electrical stimulation using H&E sections under stimulated retinal zones at 749 μC/cm^2^/ph showed a series of bloated eosinophilic punctate processes suggestive of the swelling of the end feet of Müller glia (Cohen et al., 2011). Figure 3 shows examples of a rabbit retina wholemount area stimulated at 749 μC/cm^2^/ph, imaged with OCT and stained green with an anti-GS antibody. Unlike human retina, the rabbit retinal GCL is of low cell density, and is filled in with many Müller cell endfeet (Oyster et al., 1981). In the unstimulated condition, the OCT B-scan cross sectional image of the retina under the stimulation electrode tube showed little change compared to the adjacent retina after 5 min (Figure 3A). In contrast, 5 min after we delivered pulse train stimulation at this charge density, the retinal layers under the electrode became swollen (Figure 3B). The ILM became detached from the GCL forming a bleb, and swelling was seen in the GCL. In the outer retina underlying the electrode edges, there is retinal detachment from the RPE. An OCT comparison of the retinal layer reflectivity after stimulation is seen in Figure 3C. Here the ILM line is translocated vertically and a large increase in seen in the reflectivity of the IPL. In addition the outer retina is also swollen by stimulation and some disruption of the outer segment layers is seen. A similar swelling pattern was seen in all 5 retinas tested at this charge density (see also Cohen et al., 2011).

After stimulation, the same OCT-imaged retinal zone was stained using antibodies against GS which labeled Müller processes green and optically sectioned using confocal microscopy at several depth levels in the inner retina below the electrode (Figures 3D–F). The insets show 2× enlarged retinal regions inside and outside the stimulation zones at several levels in the inner retina. At the retinal surface (0 μm) below the ILM, the unstimulated rabbit retina is filled with the small block-like endings of Müller cell endfeet (Figure 3D). In contrast under the stimulus electrode, a ring-like disruption of Müller cell immunoreactivity is seen (R), and the central region under the electrode is dark (hypofluorescent). However, ganglion cell nerve fibers still cross the inner retinal surface (arrows) suggesting that this dark image in the stimulated center is due to a loss of GS immunoreactivity, and not an artifact of tissue loss during immunocytochemical processing. Focusing the lens further down at the level of the ganglion cell somata (−10 μm), the ganglion cell somata are surrounded by the Müller cell endfeet processes (Figure 3E and inset). In contrast in the stimulated lesion zone, the GS immunofluorescence is disrupted near the edges of the electrode (R), while distinct ganglion cell nuclei can still be seen as blue dots under the central region of the electrode (shown in the inset by arrowheads). In the central zone, there is again a loss of GS immunoreactivity. When the objective lens focus is just above the inner nuclear layer, the GS antibody stains the vertical processes of the Müller cells outside the stimulation zone as a series of numerous fine punctate dots (Figure 3F and inset). In contrast, in the electrode stimulation zone there is a complete loss of GS immunoreactive vertical processes under the electrode edges in a ring pattern (R), while the central zone shows some weak green GS immunoreactivity, and a few Müller cell somas are visible on one side. At 749 μC/cm^2^/ph, pulse train electrical stimulation of the retina appears to cause a severe loss of GS immunoreactivity in the processes of the Müller cell endfeet directly under the electrode.

To understand how different levels of inner retinal stimulation affected the Müller glial cells, we had to compare how these stimulus charge densities imaged using OCT cross sectional retinal B-scans altered the retinal structure. OCT is an imaging technique that can detect fine structural changes to the living retina. Electrode stimulation was performed at close proximity to the retinal surface and the different charge density pulsed retinal zones were then compared in OCT B-scans to their changes in Müller cell immunostaining as seen by changes in fluorescent immunoreactivity to GS. In our previous study using electrical stimulation we found that stimulation at 749 μC/cm^2^/ph caused increases in both the retinal thickness and also hyperreflectivity in the IPL (Cohen et al., 2011). Using time-lapse OCT imaging, we stimulated 4–5 different zones on the retinal surface at 15 min intervals at charge densities of (133, 230, 442 and 749 μC/cm^2^/ph) in each experiment. Each stimulation set had a high charge density value lesion (749 or 442 μC/cm^2^/ph) to assist with registration of the immunolabeled stimulation zones in the retinal wholemount after processing using anatomical landmarks and the ophthalmoscope images. In addition we also OCT imaged the retinal surface under inactive stimulus electrodes (*n* = 7), to control for any anoxia induced damaged due to the stimulation tube being near the retinal surface but there was little change in the GS immunoreactivity.

**Figure 4:**
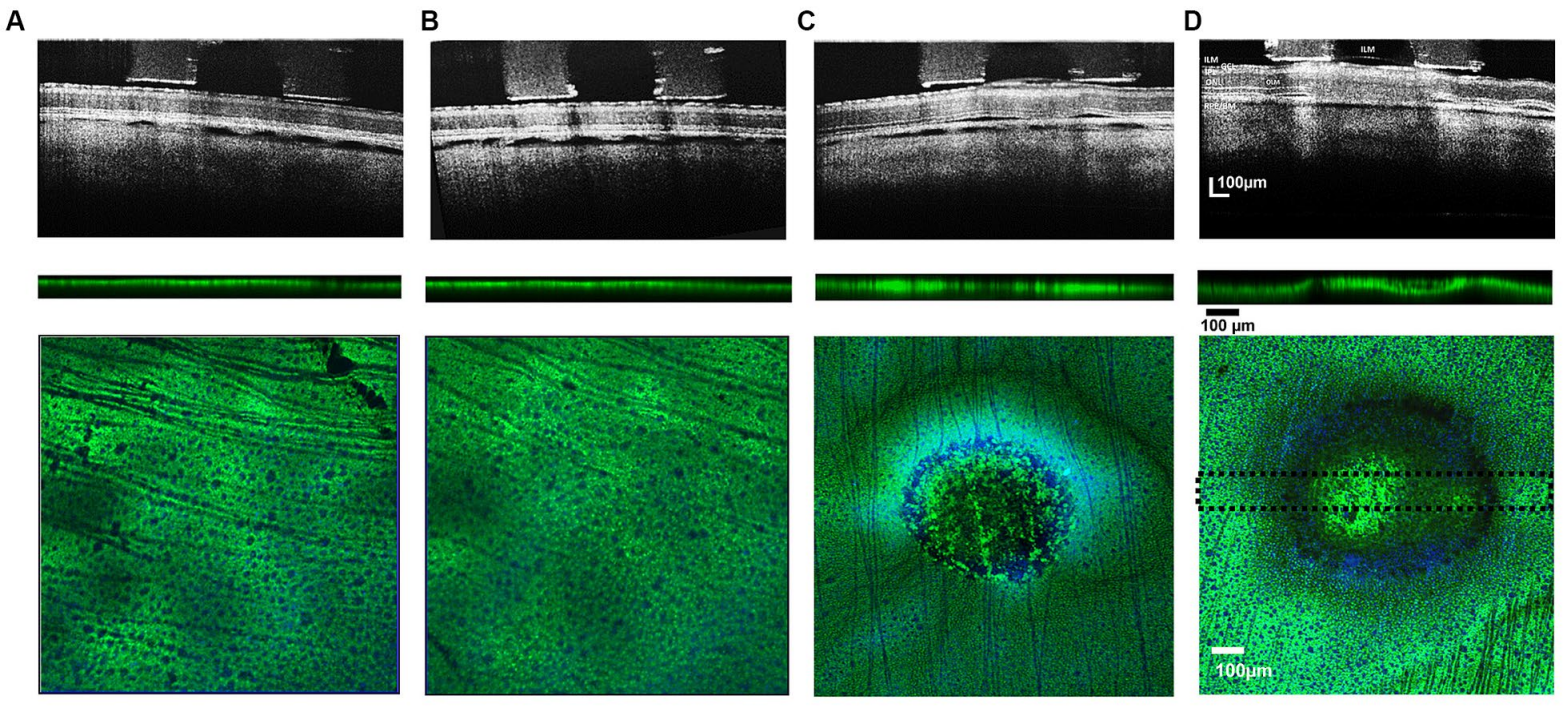
Comparison of cross-sectional b/w B-scan OCT images of stimulated retinal eyecup zones at 133–749 μC/cm^2^/ph pulse train charge densities (upper row) and their respective post-processed confocal image stacks (lower row) showing green GS-immunoreactivity of labeled Müller cells. Pulse train charge densities. **(A)** 133 μC/cm^2^/ph, **(B)** 230 μC/cm^2^/ph, **(C)** 442 μC/cm^2^/ph, and **(D)** 749 μC/cm^2^/ph. When the stimulation charge density level caused a detachment of the ILM in the OCT image, there was a concomitant loss of Müller cell GS (dark) immunoreactivity in the normally green processes in the lesion below the stimulus electrode: upper row. OCT images gathered 5 min after ending the test stimulation pulses. Middle row: Cross sectional summed 100 μm thick confocal stack image showing GS immunoreactivity through the midsection of the stimulation zone (dotted lines show thickness of analysis zone in lower image. Blue DAPI stain is not present. Lower panels, show confocal Z-stack sum of each test stimulation region with anti-GS (green) and DAPI (blue) nuclear counterstain showing underlying ganglion cell nuclei. While no noticeable damage was observed for 230 and 133 μC/cm^2^/ph pulse train stimulation **(A,B)**. Strong hypofluorescent lesions in the GS immunoreactivity pattern of the Müller cells can be observed in the 749 μC and 442 μC/cm^2^/ph pulse trains confocal images with a ring-like pattern near the electrode edge **(C,D)**. ILM, inner limiting membrane; GCL, ganglion cell layer; IPL, inner plexiform layer; ONL, outer nuclear layer; OLM, outer (external) limiting membrane; RPE/BM, retinal pigment epithelium/Bruch’s membrane).

We compared the cross-sectional B-scan OCT images of retinal eyecup stimulation zones and the GS immunoreactivity pattern staining of Müller glia seen by confocal microscope reconstruction as shown in the montage of Figure 4. The upper panel shows OCT images of the retinal cross section under the electrode 5 min after stimulation by different charge density pulse trains of (133–749 μC/cm^2^/ph), while the middle and lower panels show the corresponding GS fluorescent green immunolabeling in the confocal microscope cross section (middle) and in the summed Z-stack of confocal images of the retinal surface under the stimulation zone.

At low levels of stimulus pulse trains at a charge density of 132 μC/cm^2^/ph, there was little change in the retinal structure as seen in OCT-B scans of the retina during the stimulus pulse trains (Figure 4A). In the confocal microscope, the field of GS immunostained Müller endfeet under the retinal region stimulated with the electrode appeared unaltered in either vertical cross sections or summed Z-stack surface images (5/5 cases).

For pulse trains at 230 μC/cm^2^/ph, there was also little or no alteration of the retinal structure in the OCT image in most (6/7) cases, and the GS immunolabeling did not reveal any circular lesions (Figure 4B). However in one case at 230 μC/cm^2^/ph stimulation caused swelling in the OCT image. In the confocal microscope, when this anti-GS stained zone was analyzed there was a distinct circular zone of Müller endfeet disruption, and a concomitant loss of immunoreactivity of the Müller cell processes (data not shown). No ring lesion was observed.

However, at stimulus charge densities of 442 μC/cm^2^/ph or greater, there was always swelling in the retinal structure in the OCT B-scan image in 7/7 cases (Figure 4C). The ILM became detached, and the GCL and the inner plexiform layer began to swell. In addition, there were increases in the OCT reflectivity of the inner plexiform layer. OCT B-scans also showed retinal detachments near the electrode edges in the subretinal space. In the confocal microscope at the inner retinal surface, the GS immunoreactivity pattern always showed a diminished fluorescent green pebbled pattern over the electrode center. At the electrode edge, a ring-like severe loss of GS immunoreactivity was seen at the inner retinal surface in 5/7 cases (See also Wiley and Webster, 1982). Deep below the inner retinal surface, the regular punctate staining of the field of Müller cell endfeet processes was lost under the whole electrode lumen area in 7/7 cases.

After the highest level of stimulation 749 μC/cm^2^/ph, there was a strong hyperreflectivity in the underlying ILM-IPL region of the OCT image commonly termed the GCL, which contains many Müller cell endfeet (Figure 4D). As stimulation continued, the ILM basement membrane often separated from the swollen GCL with the subsequent formation of a bleb-like structure in 5/5 cases. There were retinal detachments near the electrode edges in the subretinal space. At the inner retinal surface, the GS immunoreactivity pattern showed a less pebbled green blob like pattern over the electrode center in 4/5 cases while in one case almost all the GS center staining near the surface was lost. Only nerve fibers were seen. A dark ring-like loss of GS immunoreactivity was seen at the retinal surface near the electrode edge extending deep into the retina in all 5/5 cases examined. Again, the regular punctate anti-GS staining of the Müller cell processes was lost under the electrode lumen in all cases, which when summed, left an indistinct green central spot.

**Figure 5:**
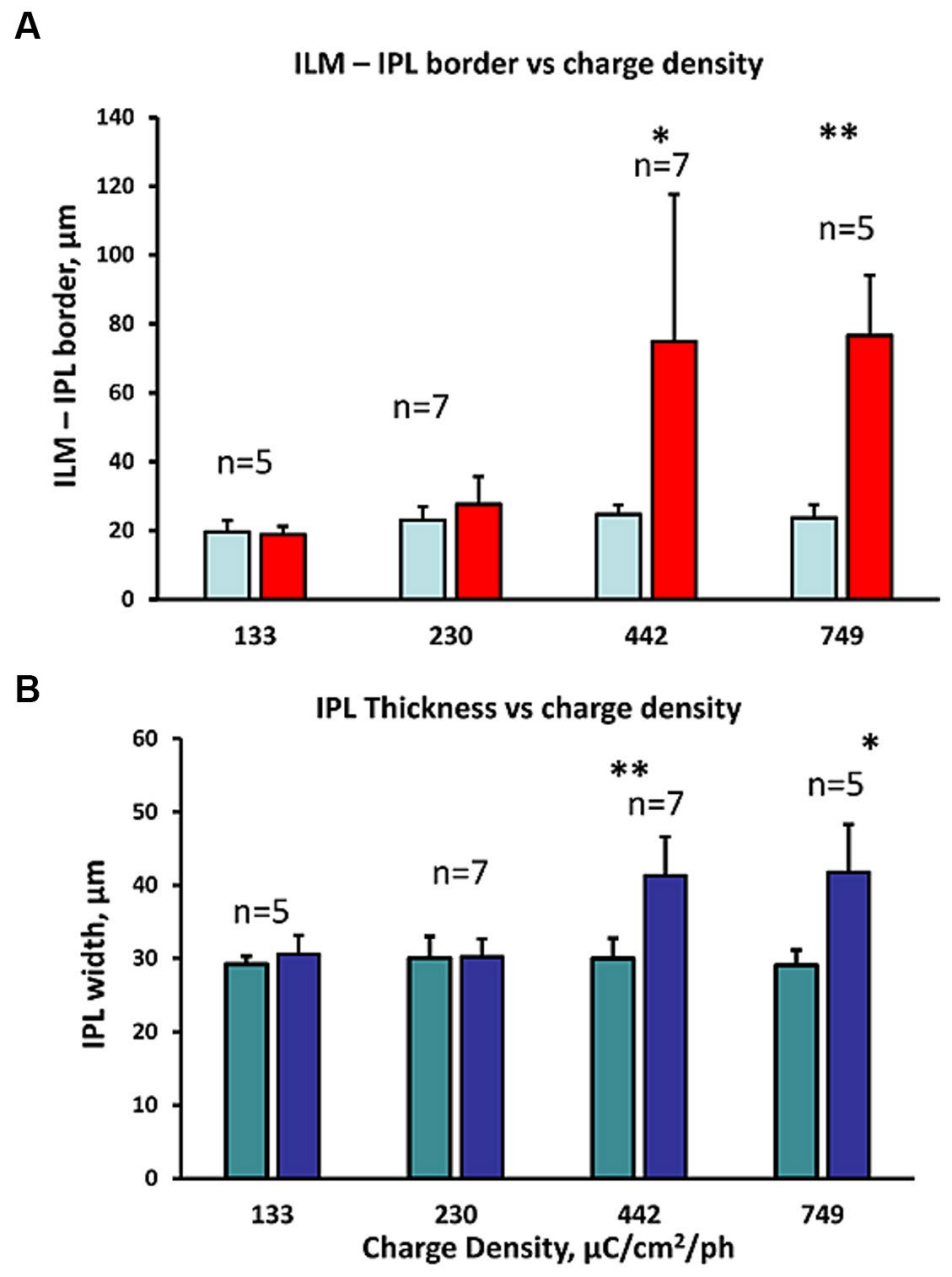
Comparison of the inner retinal layer swelling metrics observed in OCT B-scans at different charge densities (132–749 μC/cm^2^/ph). **(A)** Comparison of the IPL border to ILM width distance in the OCT B-scan image before and after stimulation (right/left bars respectively). The charge density of the pulse trains tested are shown below. Large ILM-IPL thickness changes reflect the detachment of the ILM often observed with bleb formation in the OCT image during high (442–749 μC/cm^2^/ph) pulse charge density train stimulation. *p < 0.05, **p ≤ 0.005 significance (2 sample paired *t*-test). **(B)** Comparison of the change in IPL thickness in the OCT B-scan image at different charge density pulse trains tested (shown below). *p ≤ 0.01, **p ≤ 0.005 significance (2 sample paired *t*-test). Again, IPL swelling was seen at the highest 2 charge densities tested.

We measured the swelling in the GCL in the OCT images before and after increasing charge density levels of retinal stimulation. The average change in the ILM-IPL border width with different charge densities of stimulation is shown in Figure 5A (see inset to Figure 1 for method). At low charge densities (133–230 μC/cm^2^/ph), there was little change in the ILM-IPL border width, while at 442–749 μC/cm^2^/ph, the ILM-IPL border increased significantly, being 3.0, and 3.35× controls, respectively, as the ILM detached from the retina (*n* = 7, 5 zones respectively). This ILM detachment and bleb formation seemed to suggest some form of fluid build up was being generated during excessive stimulation levels. However, we note the swelling of the ganglion cell layer (mainly Müller cell endfeet in rabbits) was often less than the distension of the ILM. At lower charge densities 133–230 μC/cm^2^/ph, there was little swelling or injury to the Müller cell endfeet. The average inner plexiform layer width behaved in a similar manner to pulse train stimulation at different charge densities. At 133–230 μC/cm^2^/ph levels of retinal stimulation there was no effect. In contrast at 442–749 μC/cm^2^/ph, the OCT images of the inner plexiform layer were significantly swollen under epiretinal stimulus electrodes (Figure 5B). On average pulse train stimulation at 442–749 μC/cm^2^/ph significantly increased the IPL width 38% and 43% in OCT images, respectively.

**Figure 6:**
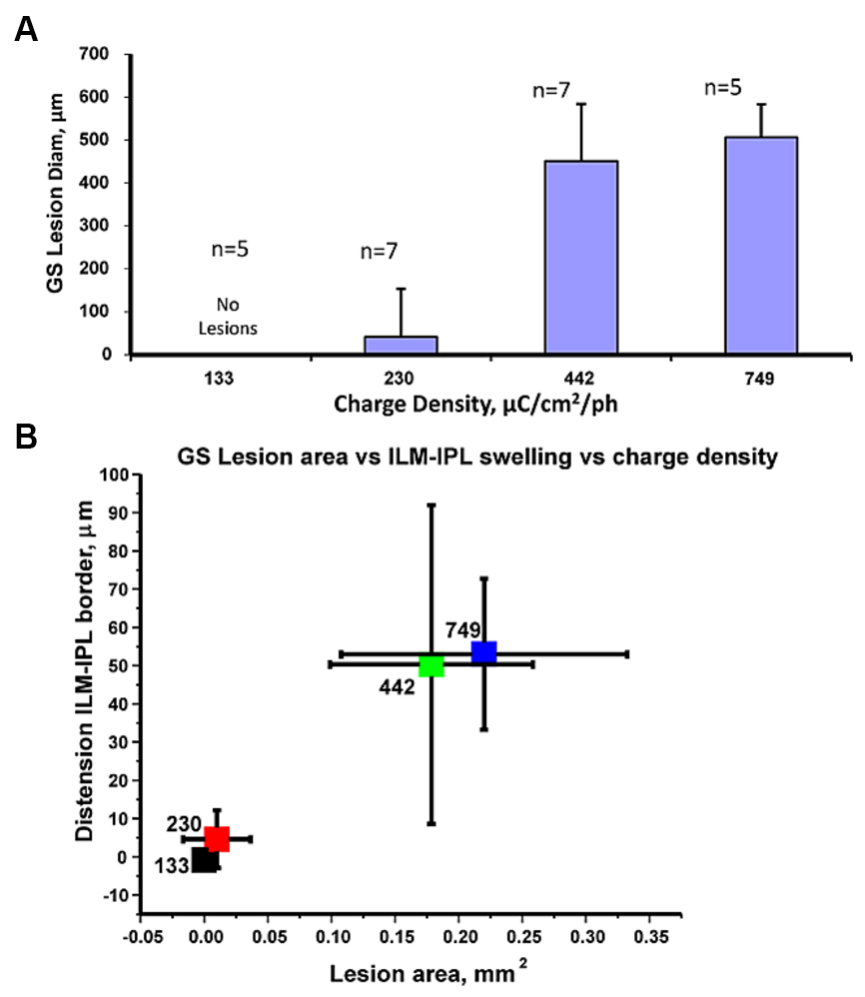
**(A)** Comparison of the average GS lesion diameter vs. the stimulus pulse charge density delivered to the retinal surface. Statistics: Kruskal–Wallis tests showed significant differences in the data values. **(B)** Cluster plot comparing mean change in glutamate synthetase immunostained lesion stimulated area on the retinal surface vs. mean relative change in ILM distension (from IPL border) at the 4 charge densities tested. The pulse charge density in μC/cm^2^/ph for each level is indicated on individual markers. Error bars denote standard deviations. The high pulse charge density clusters (749,442 μC/cm^2^/ph) are separate from those at 233, 132 μC/cm^2^/ph. Sample numbers as previous figure.

The GS immunoreactivity pattern in retinal stimulation zones in the retina showed a similar pattern where, high charge density stimulus pulses that caused inner retinal swelling seen with OCT appeared to be correlated with a loss of GS immunoreactivity in the Müller cell endfeet. As shown in Figure 6A we plotted the average diameter of the GS lesion found in stained retinal wholemounts with the pulse train charge density tested. At low stimulus *pulse* charge densities (132–230 pulse μC/cm^2^/ph), there were few or no lesions in the GS immunostaining of the stimulated retinal surface, while 442–749 μC/cm^2^/ph caused extensive lesions. The average diameter of the observed GS stained lesions at 749 μC/cm^2^/ph was 521 ± 105 μm (mean ± std. dev.) *n* = 5. These glutamate synthetase lesion zone areas at the different charge densities tested were significantly different, using a non-parametric Kruskal–Wallis statistical test of the 4 zones (*p* < 0.0002).

We examined if the swelling of the inner retina measured at a stimulation zone with OCT at a given charge density was correlated with the same zones Müller cell GS immunoreactivity loss. A cluster plot comparing the average GS lesion area to ILM-IPL border measurement changes showed distinct non-overlapping clusters as shown in Figure 6B. Although there was some variability, the swelling of the ILM-IPL border seen in OCT images and the area of the Müller cell lesion zone appeared to form distinct clusters. Low charge density clusters (132–230 μC/cm^2^/ph) with little or no GS lesion area, had little change in the IPL-ILM border after stimulation and did not overlap with (442–749 μC/cm^2^/ph) clusters which had significant GS lesions and IPL-ILM dimensional changes. Given the separated clusters of the IPL-ILM swelling with the loss of synthetase immunoreactivity at high charge density values (442–749 μC/cm^2^/ph), this raised the question how much stimulus current by an epiretinal electrode normally passes through these radial Müller glia processes whose endfeet cover the inner retinal surface.

We examined what contribution transretinal stimulation currents generated by external electrodes traverse through open conductance channels in the vertical pathways of the normal retina in the eyecup preparation. We examined the hypothesis inner retinal stimulus current conductances could propagate radially in the oriented Müller glia as their processes display an inward rectifier potassium conductance that is active at rest on both their processes endings in the inner (endfeet) and external outer retina. Alternatively, the transretinal current could propagate through the retinal interstitial spaces. Many physiological studies have shown Müller glia display a dominant potassium conductance at rest on the endfeet that is highly sensitive to blockade by as little as 200 μM barium in the Ringer (Newman, 1986; Reichelt and Pannicke, 1993; Chao et al., 1994; Solessio et al., 2000).

To examine what role resting potassium conductances from ion channels played in conducting these vertical stimulus electrode currents across the retina, we delivered periodic transretinal current pulses across the eyecup preparation using Pt electrodes and monitored the voltage drop across the eyecup using a low resistance saline microelectrode at the retinal surface (see Methods for details). This current generates a two component eyewall charging curve (Tomita et al., 1960; Faber, 1969). It is generally believed that the slow RC charging current components reflects the time constant of the capacitance of the highly infolded membrane of the pigment epithelium and its resistive junctions (e.g., Rodieck, 1973, Ch. 18), while the fast onset voltage step is thought to reflect the rapid combined resistive voltage drop across the retina and the back eye layers (i.e., choroid/sclera) (e.g., Faber, 1969).

**Figure 7:**
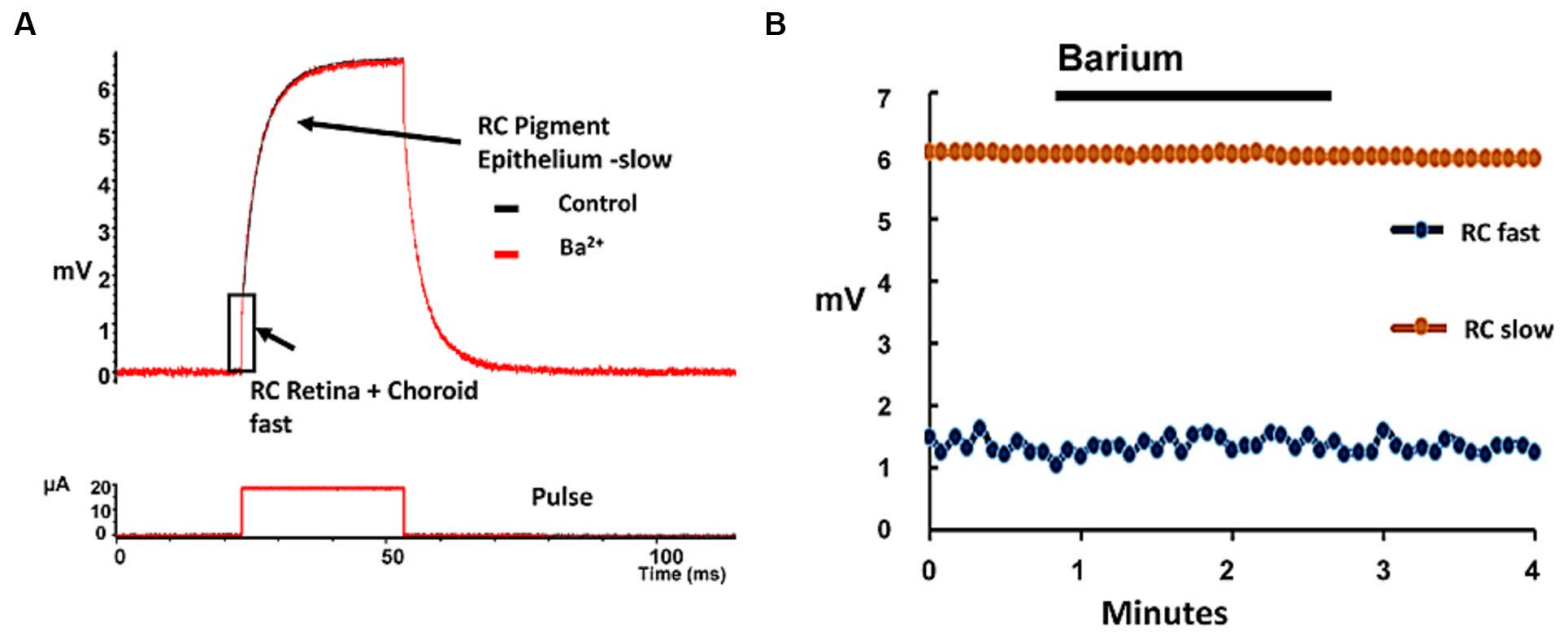
Addition of barium, a potassium channel blocker had only minor effects on the eyecup resistance to transretinal currents. **(A)** Example of the voltage charging waveform of the rabbit eyewall (upper trace) in response to a transretinal current pulse (lower trace) across the eyewall layers in the control condition (black trace) and in the presence of 2 mM barium (red trace). The fast transient voltage RC component (rectangle) represents the change in transretinal eyewall resistance (retina/choroid/sclera), while the slower component is thought to reflect mainly the charging of the pigment epithelium (arrows). **(B)** Plot of the change in the charging properties of the two RC time constants of the eyewall during the application of barium (2 mM). Each point denotes a time constant measurement of the eyewall in the eyecup preparation. Little change was seen in either the fast RC (blue dots, denoting retina + choroid/sclera) or slow RC (orange dots, denoting RPE) charging curve amplitudes components in barium.

We passed small 20 μA transretinal currents across the eye-wall tissue to examine the normal eyecup charging curve kinetics (Figure 7). When currents are passed across the living rabbit eyecup, this slow charging time constant component was found to average 4.01 ± 0.27 msec (mean, s.d. *n* = 5 retinas). In contrast the fast charging current step component had a time constant of 0.41 ± 0.02 msec (mean s.d. *n* = 5). On average application of Ringer containing 2 mM barium, a potassium channel blocker caused little change to the fast charging component resistance amplitude averaging 97.8 ± 3.5% of control values (mean, s.d. *n* = 5) suggesting it had a small effect on the trans eyecup retinal resistance. In addition, in barium, the amplitude of the slow TC of the RPE was also unaffected averaging 107.3 ± 13.0% of control values (mean, s.d *n* = 5) (see Tao et al., 1994). These negative results would seem to suggest in the normal retina during modest stimulus current pulses, the barium-sensitive potassium channel conduction pathway may play only a minor role in transretinal current flow across the retina during low levels of electrical stimulation (<233 μC/cm^2^/ph) by epiretinal electrodes (see also Discussion).

### 3.2 Astrocytes

We used an antibody against glial fibrillary acidic (GFAP) protein to rapidly label the astrocyte intermediate filaments. While this antibody is commonly used to study the upregulation of glial fibrillary acid protein (GFAP) expression as an indicator of Müller cell gliosis, the upregulation of GFAP typically requires waiting 2–12 days post-injury (e.g., Cao et al., 2001; Colodetti et al., 2007). However, after 4 h after electrical stimulation, we observed no increase in GFAP immunoreactivity in the stimulus regions with high pulse charge density lesions imaged using OCT (see Supplementary Figure S1A). Similar to previous reports in the rabbit retina, our anti-GFAP antibody showed labeling that was restricted on the retinal surface to astrocytes in the medullated nerve fiber bundles of the optic radiations (Schnitzer and Karschin, 1986). Attempts to stain Müller cells with GFAP waiting post stimulation up to 4 h were negative (*n* = 8 zones) (Supplementary Figure S1B). Since the majority of the rabbit retina surface did not contain these astrocytes, our analysis of pulse train stimulation on these glial cells was limited.

### 3.3 Microglia

The final common glial cell potentially sensitive to high charge density pulse train stimulation injury are the inner retinal microglia. In the rabbit, many microglia processes arborize in the inner retina just below the stimulation electrode. In our initial experiments, we attempted to label the field of inner microglia for time lapse study by intraocular injection of a fluorescent lectin (see Methods for details). An example of a movie of live rabbit microglia that were prelabeled in the eye using the Griffonia lectin in the retinal eyecup is shown in the Supplementary Video 1.AVI. Time lapse confocal imaging showed their unstimulated terminal processes were highly active (*n* = 4 retina movies). These results indicated that microglia could be labeled by intraocular injection of lectin in the live animal. However, after enucleation and an hour of eyecup imaging, the lectin labeling was often uneven on the retinal surface and the microglia process labeling tended to become patched; hampering time-lapse tracking analysis of microglia. Hence all fluorescent lectin labeling of stimulated retinal zones microglia was done by bath incubation of lectin in the eyecup post stimulation.

**Figure 8:**
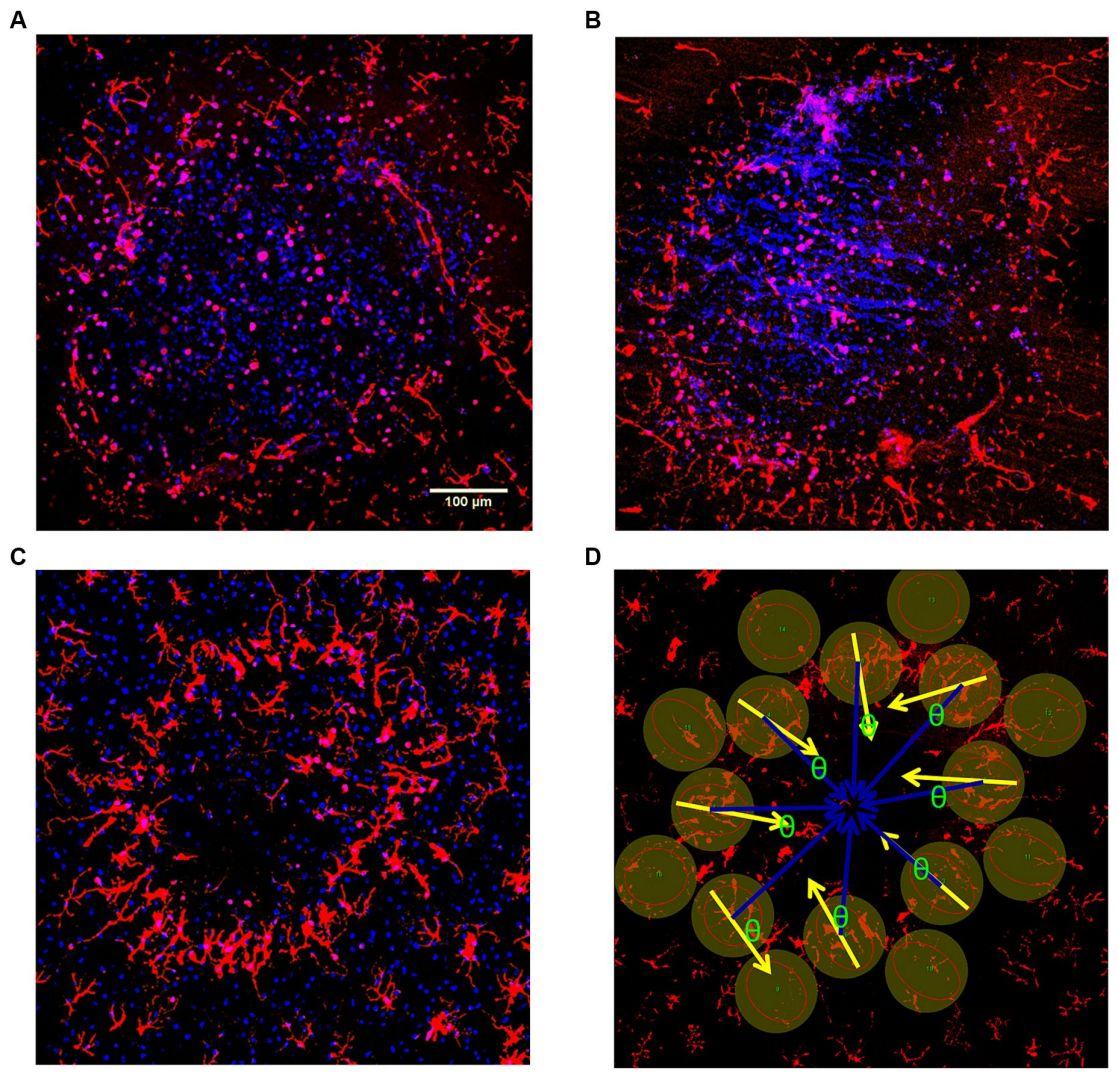
**(A–C)** Confocal Z-stack examples of response of the rabbit retina inner microglia response to damaging high pulse charge density train stimulation (749 μC/cm^2^/ph) at different times post-stimulation. The retinal microglia in the retina (red) were confocally reconstructed in each stimulation delay zone. DAPI was used as the nuclear counter stain (blue) which helped allow identification of rounded microglia and delineate the lesion edges. **(A)** Confocal Z-stack image (0 hr) just after stimulation (0 hr) Inside the lumen, the microglia appear severely rounded. There is little process orientation outside the lesion. **(B)** Confocal Z-stack image 1 hr after stimulation. **(C)** Confocal Z-stack image 3 hr after stimulation, shows microglia beginning to orient towards the lesion, and congregate at the lesion edge (ring). **(D)** Confocal Z-stack image 4 hr after stimulation showing method of microglia process orientation measurement. DAPI stain is not displayed. The cosine of the angle difference (θ) between the measured orientation (yellow arrows) and the ideal central orientation (blue arrows) were analyzed and weighted by coherence for each lesion (see Methods for details).

We examined post stimulation how the retinal microglia reacted morphologically with time to pulse train stimulation of the overlying retina at a high charge density (749 μC/cm^2^/ph). We stimulated the surface at different times with up to 5 spatially separate lesion zones on the retinal eyecup surface. Stimulation time points at 0, 0.5, 1, 2, 3, and 4 h post stimulation were studied in *n* = 5, 3, 5, 5, 5, and 3 zones, respectively. The eyecup was then lectin stained to label the zones microglia morphology post stimulation followed by aldehyde fixation. This method resulted in more uniform labeling of the whole retina. A total of 26 spatially separate retinal lesion zones were analyzed at different time points after stimulation. Just after stimulation (0 hr), the microglia processes inside the central zone under the electrode appeared contracted and circular, which can be seen as a series of small red dots in summed confocal Z-stack images (Figure 8A). Outside the lesion, there is little microglia process orientation. By 3–4 h post electrode stimulation, the microglia appeared to congregate around the retinal lesion edge, orient inwards, and their processes began to thicken (Figures 8C,D).

**Figure 9:**
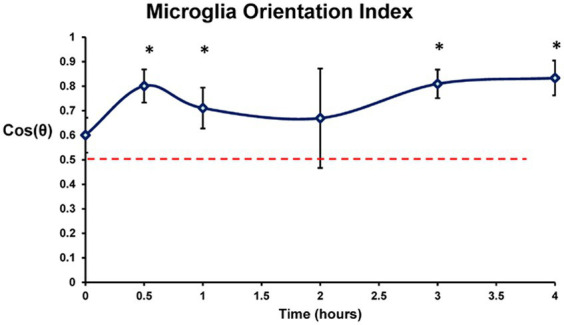
Orientation response of the lectin labeled retinal microglia in the confocal Z-stack images plotted by time post stimulation (749 μC/cm^2^/ph). Orientation in each stimulated zone was scored relative to the center of the stimulated lesion and weighted by coherency. Dotted line denotes orientation index of randomly oriented microglia. Completely random orientation would average out to 0.5 (0 being completely unaligned and 1 perfectly aligned). 0 h, 0.5 h, 1 h, 2 h, 3 h, and 4 h post stimulation were studied. Mean ± s.d. * indicates significant differences with 0 h (2 sample paired *t*-test, *p* < 0.05).

The effect of electrode stimulation on the orientation of the microglia outside the high charge density stimulation zone was analyzed in groups of 8 microglia cells around each electrode zone at each stimulation time point. The cosine of the angle difference (θ) between the measured microglia process orientation (yellow arrows) and the ideal center radial orientation (blue arrows) were analyzed and weighted for coherence for each lesion. After stimulation period delays of 30 min or more prior to staining, the microglia processes just outside the stimulus electrode lumen began to orient around the stimulation zone pointing inwards in many cases. Using an orientation analysis program on the microglia morphology, our results indicated there was significant outer microglia process orientation into the stimulation zone within 30 min post-stimulation, reaching an index of 0.7–0.8 which persisted for 4 h (Figure 9) (2 sample paired *t*-test vs. 0 h. delay, *p* < 0.05) compared to population fields of non-oriented cells.

## Discussion

We have examined the effects of electrical stimulation on the glial support cells of the rabbit retina at different charge densities (133–749 μC/cm^2^/ph). The rabbit retinal eyecup *ex-vivo* model allows real-time optical comparison of the effects of electrical stimulation by transparent electrodes on a securely mounted eyewall at a controlled electrode proximity (30 μm) away from the retinal surface. This *ex-vivo* preparation avoids the instability issues of *in-vivo* preparations where if not properly stabilized, any slight animal movement can confound physical electrode damage to the retina, with damage from electrical stimulation (see Ikeda and Pringle, 1971). The top levels of pulse charge densities tested here in the eyecup preparation are similar to those used in clinical testing of phosphene thresholds of Argus I and II retinal implants in human subjects: (up to 1870 μA, and were limited to charge densities of 1 mC/cm^2^/ph) (e.g., Figure 5; Supplementary Figure S2; De Balthasar et al., 2008; Figure 4; Ahuja et al., 2013, see also Xu et al., 2021). It should also be noted that phosphene stimulation thresholds in humans can vary with the stimulus electrode proximity to the retina, and the retinal degeneration disease state.

We chose to acutely examine the effects of 5 min of 50 Hz pulse train stimulation at different charge densities on the glial cells in the rabbit retina (see also Cohen et al., 2011). In toxicology studies, it is often desirable to examine the tissue acutely near the time of injury (Bolon et al., 2006; Butterwick et al., 2007; Nakauchi et al., 2007; Switzer, 2011). While other stimulation studies have examined injury to retinal neurons after a delay (2–12 days) for gliosis induction and upregulation of GFAP staining in Müller glia cells, significant retinal remodeling of the injury can occur (e.g., Colodetti et al., 2007). Currently, no studies have examined stimulation’s acute effects on these glial cells directly after the time of stimulation. Our rabbit eyecup preparation model allows acute real-time *ex-vivo* imaging of these electrical stimulation damage processes applied to different locations on a large retinal surface area (1 cm diam.).

We first examined the acute effects of stimulation at different charge densities on the endfeet of the Müller cells which are located just below the ILM and are closest to the stimulus electrode, staining the cells with antibodies to GS. GS is a soluble enzyme found involved in glutamate homeostasis in the Müller cell (Bringmann et al., 2013). We used the loss of the soluble GS immunoreactivity in electroporated Müller cell endfeet processes as the damage indicator as these processes are poorly stained with more classical nuclear DNA damage indicators such as propidium iodide. The use of internal soluble enzymes as cellular electroporation detectors has been previously described using lactate dehydrogenase; e.g., muscle (Gissel and Clausen, 2003); heart (Cheah et al., 2010); and brain (Deng et al., 2014).

The GS stained vertical processes of the Müller glial support cells form a finely spaced array in the inner plexiform layer of the rabbit approaching peak densities of >10,000 processes/mm^2^ (Robinson and Dreher, 1990). While low charge density pulse trains (133–230 μC/cm^2^/ph) caused little effect on the soluble GS immunoreactivity levels in the processes of the Müller cells, there was a significant loss of GS immunoreactivity under stimulus electrodes at high charge densities of 442–749 μC/cm^2^/ph. This was correlated with swelling at the IPL-ILM border which is lined by Müller cell endfeet in the low density GCL of rabbits in OCT images (Oyster et al., 1981). However, in humans this pulse stimulation might additionally also cause more damage to ganglion cells which are present as a continuous layer in the macula (Curcio and Allen, 1990). The average diameter of the observed GS stained lesions at high pulse charge densities was highly confined-close to the calculated mean diameter of the angled transparent stimulus electrodes (451 μm vs. our measured average of 521 ± 105 μm) (Figure 7). A study using a long term retinal injury model has shown that GS staining loss was strongly correlated with glutamate redistribution and neuronal damage in a dog glaucoma model (Chen et al., 2008). In the brain, GS has been proposed as a CSF neurotrauma marker (Halford et al., 2017).

We also observed at high stimulus pulse charge densities (442–749 μC/cm^2^ ph), the curious formation of a bleb-like structure showing separation of the ILM basement membrane from the Müller endfeet. Although the precise mechanism of this bleb formation is unclear, it is suggestive of fluid formation being confined by ILM filtration similar to other forms of retinal edema seen in OCT images (Peynshaert et al., 2019). At these stimulation levels, our previous studies showed there was significant electroporation of the underling retinal ganglion cells under the stimulus electrode with prolonged stimulation. Histological studies of overstimulated retinal regions at high charge densities previously studied using OCT showed a series of swollen eosinophilic blebs resembling the Müller cell processes furcation into many endfeet previously described in rabbit (Reichenbach et al., 1989; Cohen et al., 2011). Edema can also be seen in the expansion of the ILM-inner IPL border which previous studies in rabbit retina correlated with propidium iodide nuclear staining of the damaged ganglion cells presumably due to electroporation and IPL reflectance increases using OCT (Cohen et al., 2011). At similar charge densities (442 μC/cm^2^/ph), a 1 min period of 50 Hz electrical stimulation pulses caused complete loss of light-evoked and spontaneous ganglion cell firing for many minutes in rabbit retina (Cohen, 2009), suggesting that ganglion cells in addition to glial cells may also be damaged by high charge density stimulation. It is unclear why stimulation at high charge densities (442–749 μC/cm^2^/ph) of the epiretinal surface caused ILM detachment. It is possible fluid buildup could occur below the ILM. Jackson et al. (2003) found that fluid permeability of the ILM was restricted to molecules <4.4 KD. If this high charge density stimulation caused extracellular release of internal cellular proteins or enzymes, damage to the Müller cell ion regulation pumps and excessive edema in the inner retina from electroporation, then perhaps when the basement membrane fluid filtration of the ILM becomes clogged, it could cause ILM bleb formation.

However, the edema in Müller cell processes was only caused by high pulse charge density stimulation levels causing endfeet electroporation and swelling well above our reported ganglion cell excitation stimulation thresholds studied at close retinal proximity (Cohen, 2009). The eyewall charging components to weak 20 μA transretinal stimulus currents across the retinal tissue were not blocked by adding 2 mM barium to our bicarbonate-based Ames Ringer. Barium is a well known blocker of Müller cell endfeet potassium conductances (Newman, 1986; Chao et al., 1994). Our negative result with barium blockade of retinal potassium channels would imply that under normal conditions epiretinal stimulus electrode pulse currents at low charge densities (<233 μC/cm^2^/ph) can excite ganglion cells by passing more transretinal current through interstitial space pathways than through vertical Müller glia processes in the retina.

The microglia in the inner retina can also be affected by high pulse charge density stimulation in the rabbit eye. In this initial study we only studied the microglia response in the eyecup at a high level of stimulation, previously known to cause damage to the retina (749 μC/cm^2^/ph) (Cohen et al., 2011). We used a single 5 min, high charge density pulse train electrode stimulus on sites 0–4 h before bath application of the lectin stain at the end of the experiment. This was done to avoid incomplete microglia process staining from confounding interpretation of the morphological changes at each stimulation site on the retina (see Methods). We found process orientation of the microglia outside the lesion zone began at 30 min post stimulation, the shortest time we examined. The same process orientation index was maintained when microglia were studied with longer delays after pulse train stimulation. It is also possible that with this severe damage more microglia outside the lesion zone might accumulate at the ring-like lesion edges with time.

Real-time imaging studies of transgenic mice microglia labeled with green fluorescent protein (GFP) showed focused laser injury in CNS and retinal tissue caused their processes to become oriented centripetally and extended into small local lesions (Davalos et al., 2005; Nimmerjahn et al., 2005; Eter et al., 2008; Lee et al., 2008). Although we were unable to label microglia stably using lectins in the rabbit retina *a priori* for reliable real-time tracking post stimulation, the local orienting movements of these microglia cell processes around the stimulated zone in the rabbit retina is very similar to our previous results using real-time imaging in the CX3CR1 GFP transgenic mouse retina using a smaller transparent stimulation tube electrode and the same high charge density stimulation (Yohannes et al., 2021). Here we also found, imaging up to 90 min. After stimulation, that inner retinal microglia inside the electrode were severely damaged, process rounded, immotile, and a ring-like lesion of loss of fluorescent cells was formed at the electrode edge. The initial response of microglia outside the electrode overstimulation zone edge was also process orientation centripetally toward the stimulus zone center with very limited migration internally. However, unlike injury to Müller cells, the microglia can slowly regenerate in the mammalian retinae (e.g., Zhang et al., 2018). We cannot comment on electrical stimulation’s effect on astrocytes. The rabbit retina appears to be a poor model for studies of electrical stimulation effects on the retinal astrocytes due to their limited distribution in the optic radiations.

In animal experiments, low stimulus pulse charge density thresholds exciting ganglion cells can be obtained when the stimulus electrodes are held against the inner retinal surface (e.g., Jones et al., 2015), while in implant patients the measured proximity of epiretinal stimulus array electrodes to the retina using OCT are often significantly displaced which increases charge density thresholds for phosphenes (e.g., Ahuja et al., 2013; Xu et al., 2021). The eyes of many blind patients exhibit resting nystagmus which can interfere with using OCT imaging to measure the retinal proximity of epiretinal stimulus electrode arrays (e.g., Kömpf and Piper, 1987; De Balthasar et al., 2008; Titchener et al., 2023). Our epiretinal electrode stimulation results on Müller glia injury suggest (in addition to ganglion cells) some caution should be observed on initial testing of patient’s phosphene threshold charge densities post implantation when the relative proximity of the epiretinal stimulus array electrodes to the retina has not been optically determined, particularly when using high charge density stimulus pulse trains to elicit phosphenes.

Finally, there are some differences in the glial cell densities in our rabbit retinal model and in the macular retina of primates which would be near an epiretinal stimulus electrode. Unlike primate retinas, the rabbit GCL (streak) has a low ganglion cell density, and is filled in with many Müller cell endfeet at a peak cell density of 10,700–15,000/mm^2^ (Robinson and Dreher, 1990). In the central fovea of humans, the Müller cells are reported to be present at a peak cellular density of 25,000/mm^2^, but occupy less retinal volume in the foveal region compared to the ganglion cells (Distler and Dreher, 1996; Nishikawa and Tamai, 2001; Ahmad et al., 2003). However in the human retina, ~500 μm from of the foveal center, Müller cell endfeet are reported to line the ILM similar to the rabbit retina, and have the potential to be injured by high charge density pulse trains from epiretinal stimulus electrodes (see Berdugo et al., 2021; Figure 1). In primates, microglial cells are also distributed throughout the central retina (except in the foveola) but at lower densities than Müller cells, like their distribution in the rabbit (Ashwell, 1989; Singaravelu et al., 2017). This cellular distribution presumably would result in local injury responses to overstimulation similar to those described here and in Yohannes et al. (2021). In contrast, the fine processes of inner retinal astrocytes are largely absent from the avascular rabbit retinal model, but are present at low densities in the nerve fiber and ganglion cell layer of the vascular retinas of primates and humans (Schnitzer and Karschin, 1986; Ramirez et al., 1994).

## 5 Summary

Using a rabbit retinal eyecup model, we examined the acute effects of pulse train stimulation on glial cells, which are often closest to surface stimulation electrodes. In the retina, the Müller cells, microglia and astrocytes are the glial cells that line the surfaces of the neuronal tissue and support neuron function. We compared swelling under the stimulus electrode in the OCT image to selective immunostains for retinal glia. We found high charge density stimulus pulses (442–749 μC/cm^2^/ph) by surface electrodes led to significant injury and edema to the Müller glia lining the inner retinal surface. Because Müller cells make up a significant portion of the retinal volume, poorly regenerate in higher mammals (Gao et al., 2021; Salman et al., 2021), and play key roles in the homeostasis of ions and molecules for the neuron environment, it is important we consider the effects of electrical stimulation pulses on these key support cells which in addition to neurons often lie close to the surface electrodes used in stimulating neuroprosthetic devices.

## Data availability statement

The raw data supporting the conclusions of this article will be made available by the authors, without undue reservation.

## Ethics statement

The animal study was approved by White Oak Animal Program ACUC. The study was conducted in accordance with the local legislation and institutional requirements.

## Author contributions

DH: Funding acquisition, Investigation, Resources, Writing – review & editing. JM: Formal analysis, Investigation, Methodology, Writing – review & editing, Software. EC: Formal analysis, Investigation, Methodology, Writing – review & editing, Funding acquisition.

## Supplementary material

SUPPLEMENTARY FIGURE S1 Supplemental Data: Example of an epifluorescence micrograph of a rabbit retina fixed post stimulation and incubated in anti-GFAP antibody (blue). **(A)** The astrocytes in the optic nerve radiations are labeled. **(B)** Electrode stimulation zone 4 Hrs post stimulation in an avascular region of the retina. No GFAP labeling of astrocytes or Müller cells were observed at the stimulation site (arrow) using a charge density known to cause retinal damage (749uC/cm^2^/ph).

SUPPLEMENTARY VIDEO S1 Example of time-lapse images of rabbit retina microglia labeled by Alexa 488 Isolectin B4 in the retinal eyecup preparation on the confocal microscope at 40X at 35°C. The retina was prelabeled by lectin injection into the eye (See Methods). Note the unstimulated microglia at rest are highly motile and their process are constantly probing the surrounding inner retinal tissue.
