# Supplementary figures and images for: Effect of epiretinal electrical stimulation on the glial cells in a rabbit retinal eyecup model
Source: Front Neurosci. 2024 Jan 22;18:1290829. doi: 10.3389/fnins.2024.1290829 (PMC10839094; doi:10.3389/fnins.2024.1290829)

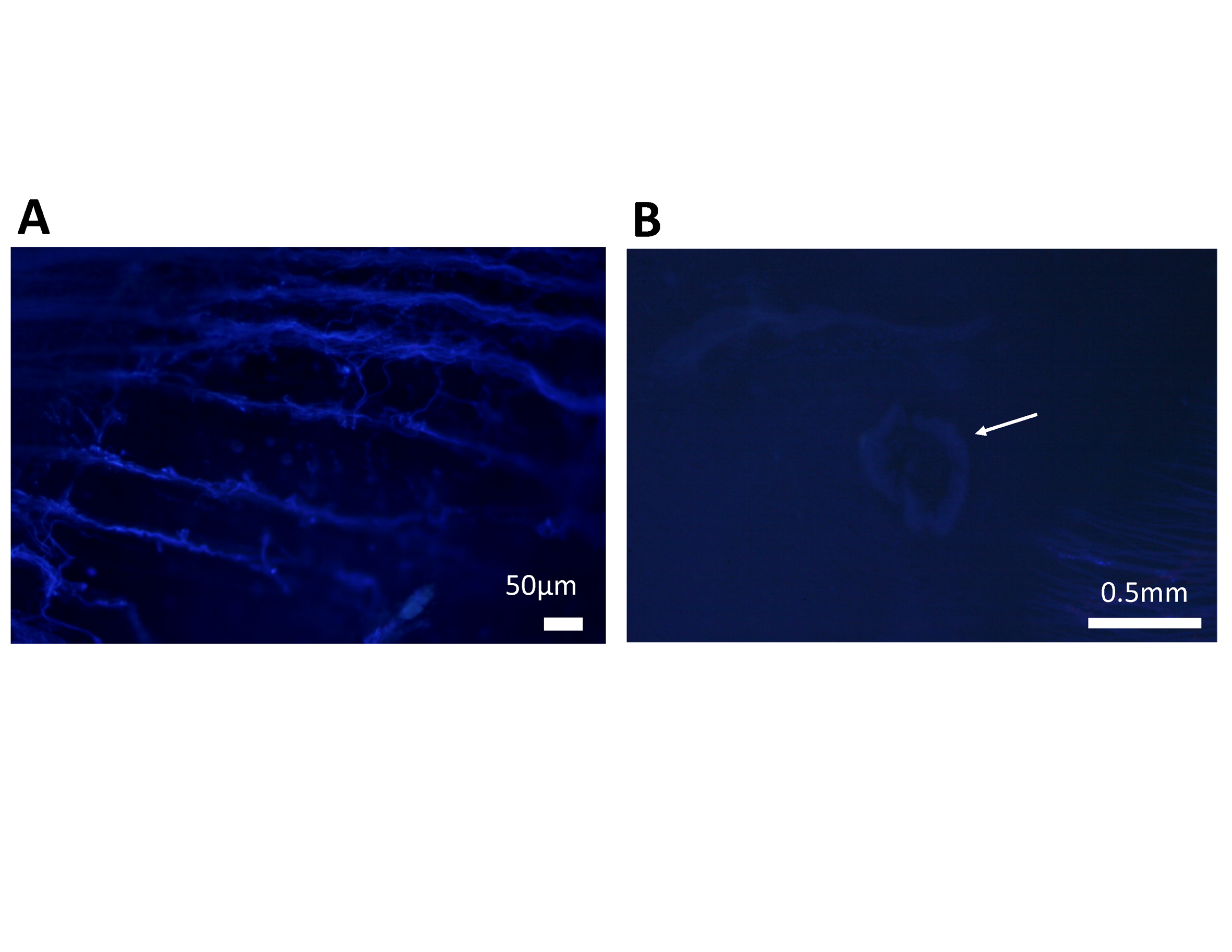

Supplement: SUPPLEMENTARY FIGURE S1 — Supplemental Data: Example of an epifluorescence micrograph of a rabbit retina fixed post stimulation and incubated in anti-GFAP antibody (blue). (A) The astrocytes in the optic nerve radiations are labeled. (B) Electrode stimulation zone 4 Hrs post stimulation in an avascular region of the retina. No GFAP labeling of astrocytes or Müller cells were observed at the stimulation site (arrow) using a charge density known to cause retinal damage (749uC/cm2/ph). [file Image_1.TIF]
